# Spectroscopy of Oscillation Modes in Homogeneously Precessing Domain of Superfluid $^3$He-B

**DOI:** 10.1007/s10909-024-03051-y

**Published:** 2024-02-17

**Authors:** V. V. Zavjalov, A. Savin, E. Sergeicheva, P. J. Hakonen

**Affiliations:** 1 Low Temperature Laboratory, Aalto University School of Science, Espoo, Finland; 2 Department of Applied Physics, Aalto University School of Science, Espoo, Finland; 3 Present Address: Department of Physics, Lancaster University, Lancaster, UK

## Abstract

We study the homogeneously precessing domain (HPD) in superfluid $^3$He-B in a regular continuous-wave nuclear magnetic resonance (CW NMR) experiment. Using Fourier analysis of CW NMR time traces, we identify several oscillation modes with frequency monotonically increasing with the frequency shift of the HPD. Some of these modes are localized near the cell walls, while others are localized in bulk liquid and can be interpreted as oscillations of $\vartheta$-solitons. We also observe chaotic motion of the HPD in a certain range of temperatures and frequency shifts.

## Introduction

Nuclear magnetic resonance (NMR) in superfluid $^3$He has played an important role in studies of its properties since the discovery of superfluid phases in 1972 [1]. The condensate in superfluid $^3$He is formed by p-wave pairing of fermions (pairs having spin 1 and orbital angular momentum 1), which allows the order parameter of the system to be written as 3$\times$3 complex matrix. A few superfluid phases with distinct broken symmetries have been observed [2]; the most studied phases are the A phase and the B phase [3]. In the B phase, the order parameter structure involves an arbitrary 3D rotation matrix which sets the mutual orientation of spin and orbital spaces. The degrees of freedom of this matrix lead to three possible spin-wave modes, strongly affected by spin-orbit interaction. As a result, many unique phenomena can be observed in NMR experiments, including signatures of non-uniform spatial distribution and topological defects in the order parameter field [4–6], longitudinal NMR [7], and homogeneously precessing domain (HPD) [10]. Fundamental connections to physics in other areas have also been established [8, 9].

The HPD was discovered in 1985 [10, 11]. Since then, it has been used as a convenient probe in experiments with spin supercurrents [12] and vortices [13]. Various oscillation modes of HPD have been studied, including uniform rotational oscillations around magnetic field [14, 15], corresponding non-uniform oscillations [16], and oscillations of the HPD surface [17–19].

One of possible 2D topological defects in $^3$He-B is the so-called $\vartheta$-soliton. It appears between two regions where the order parameter rotation matrix stays in different configurations at the minimum of spin-orbit interaction energy. In the regime of linear NMR, the analysis of the $\vartheta$-soliton is quite straightforward [5]. The solitons can also be present in the HPD state [20], but their structure is more involved. $\vartheta$-solitons in HPD have been observed experimentally in conjunction with spin-mass vortices in Ref. [21]. Recently, dynamics of a $\vartheta$-soliton in the HPD has been considered in numerical simulations [22]. In this work, we experimentally observe spatially localized HPD oscillation modes that can be compared with simulations and attributed to oscillations of the $\vartheta$-soliton, which so far have remained unidentified in experiments.

## Theoretical Background

In the B phase of superfluid $^3$He, broken symmetries of the order parameter can be described by a $3\times 3$ matrix

1 $$\begin{aligned} A_{aj} = R_{aj}({\hat{\textbf{n}}}, \vartheta )e^{i\varphi }, \end{aligned}$$

where $R_{aj}$ is a rotation matrix with axis $\hat{\textbf{n}}$ and angle $\vartheta$, and $e^{i\varphi }$ is a complex phase factor. Various spatial distributions of $\varphi$ and $R_{a j}$ are possible, as well as a few types of topological defects. Spin dynamics of $^3$He-B is described by nonlinear Leggett equations [3, 23] for spin $\textbf{S}$ and matrix $R_{aj}$:

2 $$\begin{aligned} \dot{S}_a= & {} [\textbf{S}\times \gamma \textbf{B}]_a + \frac{4}{15}\,\frac{\varOmega _B^2\chi }{\gamma ^2}\ \sin \vartheta (4\cos \vartheta + 1)\ n_a - \nabla _k J_{ak},\nonumber \\ \dot{R}_{aj}= & {} e_{abc} R_{cj} \Big (\frac{\gamma ^2}{\chi } \textbf{S} - \gamma \textbf{B} \Big )_b. \end{aligned}$$

Here $\gamma$ is the gyromagnetic ratio of $^3$He, $\textbf{B}$ denotes the external magnetic field, $\chi$ is the magnetic susceptibility, $e_{abc}$ is a permutation tensor, $J_{ak}$ specifies the spin current which carries component *a* of spin in the direction *k*, and $\varOmega _B$ is the Leggett frequency, a measure of spin-orbit interaction strength in superfluid $^3$He. All relaxation terms are neglected for simplicity.

Homogeneously precessing domain is a solution of these nonlinear equations [11]. Consider a coordinate system having axis $\hat{\textbf{z}}$ along the magnetic field, and rotating around this direction with some frequency $\omega \ge \gamma B$. Then, the HPD state is given by

3 $$\begin{aligned} \textbf{n} = {\hat{\textbf{y}}} ,\qquad \cos \vartheta = -\frac{1}{4} - \frac{15}{16}\,\frac{\omega (\omega -\gamma B)}{\varOmega _B^2} ,\qquad \textbf{S} = \frac{\omega \chi }{\gamma ^2} \left[ {\hat{\textbf{x}}}\sin \vartheta + {\hat{\textbf{z}}}\left( \cos \vartheta - \frac{\omega -\gamma B}{\omega }\right) \right] . \end{aligned}$$

Note that this solution can have an arbitrary orientation around the ${\hat{\textbf{z}}}$ axis because the system is symmetric, but in a regular continuous-wave nuclear magnetic resonance (CW NMR) experiment, the presence of a radio-frequency field along ${\hat{\textbf{x}}}$ axis stabilizes HPD in $\textbf{n}\parallel {\hat{\textbf{y}}}$ orientation in the rotating frame. The important parameter here is the frequency shift $\delta _{\omega }=\omega -\gamma B$. Usually, the shift is small, i.e., angle $\vartheta$ is slightly bigger than the so-called Leggett angle, $\vartheta _L = \cos ^{-1}(-1/4)\approx 104^\circ$, and the spin is tilted by approximately the same angle in the direction of the axis $\hat{\textbf{x}}$. The fact that the deflection of the spin is connected with the precession frequency $\omega$ makes an HPD state stable in a non-uniform magnetic field. If some spatial gradient appears in the precession frequency, spin currents transfer magnetization and compensate the difference. As a result, homogeneous precession takes place in the whole volume where the HPD exists. In fact, the HPD behaves like a liquid which fills low-field parts of the experimental cell up to the level $\omega =\gamma B$. In the case of free precession, this level is determined by the total system energy which decreases because of relaxation. In a continuous-wave NMR experiment with a sufficient feed of energy, the level can be controlled by the pumping frequency or magnetic field.

The topology of the HPD state is more complicated than that of an equilibrium state of $^3$He-B because, in addition to the orbital order parameter distribution, one can have a non-trivial distribution of the spin. One possible 2D topological defect in this state is the $\vartheta$-soliton [20]. The minimum of spin-orbit interaction energy in $^3$He-B is achieved at the Leggett angle $\vartheta _l = \cos ^{-1}(-1/4)$, the soliton appears between two different energy minima, $\vartheta _L$ and $2\pi - \vartheta _L$. There are two characteristic length scales in the $\vartheta$-soliton in the HPD. First, a small core region of size $\xi _D\approx 10$ $\upmu$m, across which the angle $\vartheta$ changes; $\xi _D$ is referred to as “the dipolar length” because its scale is governed by the dipolar energy in comparison to gradient terms. Second, outside the core region, the magnetization varies on the length scale $\xi _\omega$ that is inversely proportional to the square root of the frequency shift [20]:

4 $$\begin{aligned} \xi _\omega = \frac{c_\parallel }{\sqrt{\omega (\omega - \gamma B)}} \end{aligned}$$

where $c_\parallel$ is the spin-wave velocity. The typical range of frequency shifts in our experiments is 10–100 Hz. This corresponds to $\xi _\omega =$0.15–0.5 mm.

An analytical calculation of $\vartheta$-solitons in HPD is quite an involved task. In Ref. [22], we have performed numerical simulations in one-dimensional geometry, to obtain the structure and dynamics of the $\vartheta$-soliton in HPD. A few low-frequency modes were identified in the simulation, and their dependencies on temperature, frequency shift, and radio-frequency field were determined. We use the simulated signatures and their characteristics to identify the measured oscillation modes in this work.

## Experiment

**Fig. 1:**
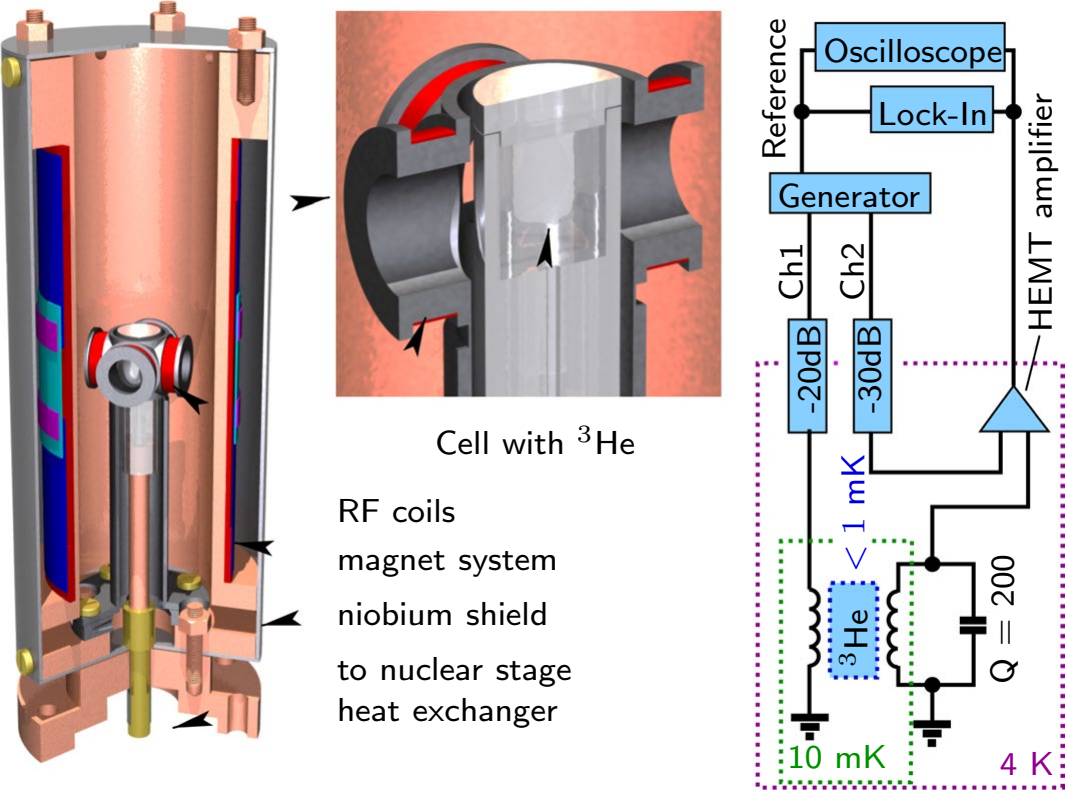
Experimental cell with NMR magnet (left), close-up of the NMR coils and the cylindrical volume (center), and schematics of the NMR spectrometer (right). Detailed description of the setup is given in the text

Our experiments were performed on a pulse-tube-based nuclear demagnetization cryostat with the minimum temperature of 0.2 mK [24]. The experimental chamber was made using epoxy Stycast-1266 (see Fig. 1). It has a cylindrical shape with an inner diameter of 7.8 mm and a length of 9 mm. The experimental volume is connected to the heat exchanger of the nuclear stage through a channel having a diameter of 1 mm in its most narrow section.

The magnet system for a uniform static field includes a solenoid with a bore diameter of 36 mm, a gradient coil and a quadratic field coil. The magnet system, thermally anchored to the mixing chamber of the cryostat, is surrounded by a superconductive niobium shield. By contact to the nuclear stage, this shield provides mechanical stabilization, while keeping thermal isolation between the magnet system and the demagnetization cooling stage. The field homogeneity in the cell volume was measured using NMR linewidth in normal $^3$He. By adjusting the current in the gradient and quadratic field coils to their optimal values, it was possible to achieve a homogeneity of $1.5\times 10^{-4}$ over the sample volume. These normal-state NMR measurements also gave us a calibration of the gradient coil: 0.157 (T/m)/A.

Our NMR spectrometer consists of two pairs of RF coils, aligned perpendicular to each other, located symmetrically at a distance of 7 mm from the central axis of the cell. These RF coils with a diameter of 12 mm are made of 50 $\upmu$m copper wire. The inductance of each coil pair is 55.5 $\upmu$H, and the calculated value of the RF field in the center is 1.66 mT/A. One coil pair is used for applying NMR excitation from a signal generator, while the other one is embedded into an *LC* tank circuit with a resonant frequency of 1.124 MHz and $Q=200$. The signal from the receiving coils is amplified by a home-made HEMT amplifier [25]. Differential input of the amplifier is used to compensate for the background signal received away from the NMR resonance. By using the excitation and compensation voltages and the parameters of the electric circuit, one can calculate currents in both coil pairs and the total amplitude of RF-field. At an RF excitation of 1 V$_{pp}$ (on the generator output), we need compensation voltage of 5.73 V$_{pp}$ which corresponds to the RF-field rms amplitude $B_{\text{ RF }}=297.6$ nT. The NMR signal is recorded by a lock-in amplifier tuned at the excitation frequency and, in parallel, by a digital oscilloscope.

Our experiments were done at a pressure of 25.7 bar in a magnetic field of 34.67 mT (the NMR frequency equals to the resonant frequency of the tank circuit). Cooling capacity of the cryostat allowed us to measure a few hours in superfluid $^3$He in each demagnetization cycle. Measurements were done both during demagnetization to the lowest temperature and while warming up.

Temperature was measured using a SQUID-based noise thermometer attached to the nuclear stage. Since it is possible to observe the superfluid transition temperature $T_c$ by means of NMR, we measured temperature difference between liquid helium and the nuclear stage at temperature $T_c$ in the sample volume. The result can be described using a simple model with a thermalization time $\tau _0$: $(T_{\text{ He }}- K\,T_{\text{ NS }})/\tau _0 = - K\,{\dot{T_{\text{ NS }}}}$, where $T_{\text{ He }}$ and $T_{\text{ NS }}$ are temperatures of the helium volume and the nuclear stage, respectively, and $K\simeq 1$ is an adjustment factor for the noise thermometer calibration. Normally, the noise thermometer is calibrated at higher temperature against a thermometer at the mixing chamber; the factor *K* fixes the inaccuracy of this calibration. Measurements at different rates of cooling give us a time constant $\tau _0=1540$ s. A rough estimation of the thermalization time using the Kapitza resistance $R_K = 900/T$ [K$^2$m$^2$/W] [26] and the normal $^3$He heat capacity $C_N=34.5T$ [J/K/mol] [27] for our amount of helium (approximately 1 mole) and sinter area (20 m$^2$) give a quite similar value $\tau _0=1550$ s.

Below the superfluid transition temperature, the Kapitza resistance is expected to follow a 1/*T* law [28], on the basis of which the thermalization time can be estimated as $\tau = C_B/C_N\,\tau _0$ where $C_N$ and $C_B$ denote the heat capacities of normal phase and B phase of $^3$He, respectively. By integrating the heat transfer model, we may estimate the temperature of $^3$He, $T_{\text{ He }}$, during the whole experiment as a function of time. The use of $T_{\text{ He }}$ significantly reduces the observed hysteresis in temperature-dependent frequency shifts measured during cool-down and warm-up, which indicates that our simple model works well.

**Fig. 2:**
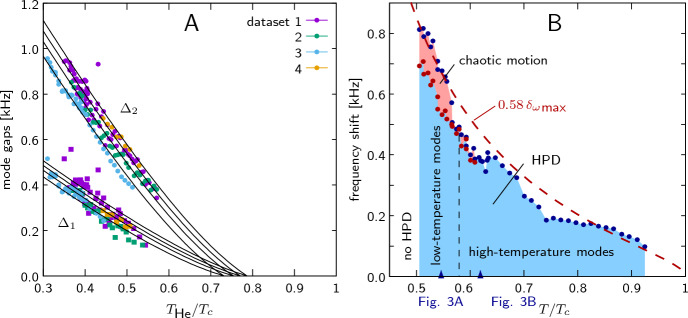
**A** Overheating correction for HPD measurements. We measure high-frequency mode gaps (will be explain later in the Results section) as a function of $T_{\text{ He }}$ temperature, assume that they are proportional to each other, and extrapolate them to zero in order to find the actual $T_c$ in the sample volume. Four different data sets are displayed, taken at different excitation levels. **B** Typical range of corrected temperatures and frequency shifts over which we detect HPD using $B_{\text{ RF }}=357$ nT (1.2 V$_{pp}$) and a field gradient of $\nabla B=-0.94\,\upmu$T/cm. The dashed red line is a theoretical curve for the maximum frequency shift using Eq. (6), with the value scaled down by a factor of 0.58 to match the measured data

There is also another thermometric uncertainty: during HPD measurements with strong NMR excitation we have a significant (about 0.2 $T_c$) overheating of the $^3$He sample. It arises because of the narrow channel between the HPD volume and the heat exchanger volume. A correction of this overheating is implemented in a simple fashion (see Fig. 2A): we determine temperature-dependent frequencies of HPD oscillations (details are given later) and assume that they should extrapolate to zero at $T_c$. We do extrapolation using a smooth rational function and obtain a temperature shift, which is naively assumed to be temperature-independent. We do this separately for each sequence of measurements, because different measurement parameters (RF-field amplitude, gradient, range of magnetic field sweep) result in different overheating. The correction has been applied to all given values of *T* except those in Fig. 2A where the uncorrected value $T_{\text{ He }}$ is used. The accuracy of such temperature corrections and our thermometry in general is quite poor, of the order of 0.1$T_c$. Nevertheless, this does not affect the main results of the work.

The HPD in our experiments was created in the usual continuous-wave method by sweeping the magnetic field in the presence of large enough RF-pumping at the resonance frequency of the *LC* circuit. The sweeping rate was always 0.807 $\upmu$T/s (26.2 Hz/s in frequency units). The appearance of the HPD could easily be observed in the NMR signal recorded by the lock-in amplifier. In parallel, we recorded the same signal by an oscilloscope to observe the response in a wide range of frequencies. Oscillations of HPD can be seen as modulation of the main signal, with side bands separated from the main signal by the frequency of the oscillations. The oscillations were visible without any special excitation. We did measurements as a function of temperature, field gradient $\nabla B$, and RF-field amplitude $B_{\text{ RF }}$. Because of overheating, we were able to create the HPD only in a small range of RF fields and only at small field gradients. Note that the field homogeneity of the NMR magnet $1.5\times 10^{-4}$ corresponds to field variations of about $5\,\upmu$T across the cell, while measurements were done at the absolute value of applied gradient from 0 to $20\,\upmu$T/cm. This means that the actual field profile and the process of HPD growth in our experiment are not well-defined. There could be a few field minima and maxima in the cell which lead to a complicated topology of the HPD surface, and this could be a possible source of $\vartheta$-solitons. However, the main results in this work are obtained on a well-defined coherent HPD that is obtained in the regime where the HPD fills the whole cell.

We were unable to observe uniform oscillations of HPD [14, 15], presumably because we could not apply a rapid step change in *B* to excite them. It would have been useful to have a separate longitudinal coil around the cell to excite uniform oscillations and use their frequency for better temperature and $B_{\text{ RF }}$ calibration.

**Fig. 3:**
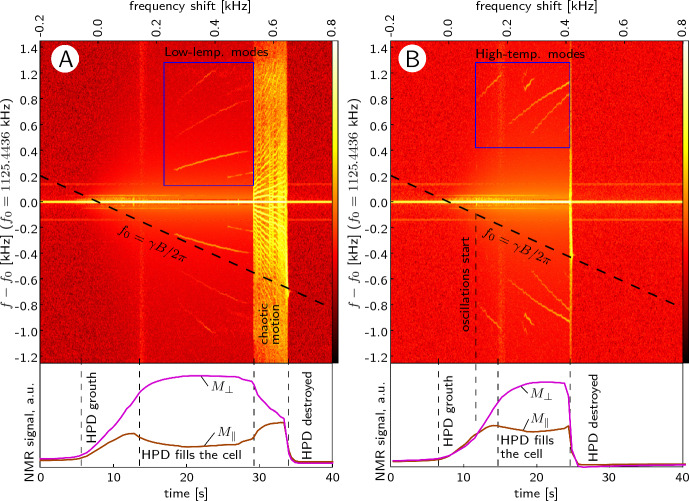
Typical recorded signals. Upper pictures display spectrograms obtained by Fourier transform on time-domain magnetization signals captured by a digital oscilloscope. The vertical axis is the frequency (difference from constant NMR frequency $f_0$), horizontal axis is time (bottom axis) or frequency shift (upper axis), which is changing because of constant ramping of magnetic field, below them are signals measured with lock-in amplifier at the excitation frequency. **A** $T=0.54\,T_c$, HPD with chaotic region and “low-temperature modes” (side bands marked with a blue frame). **B** $T=0.62\,T_c$, “high-temperature modes” (marked with a blue frame). Data in A and B were taken at pressure 25.7 bar, $B_{\text{ RF }}=357$ nT, and $\nabla B = -0.94$ $\upmu$T/cm

**Fig. 4:**
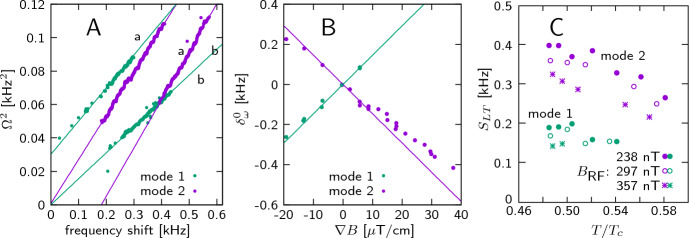
Characteristics of low-temperature modes. **A** Example of mode frequencies as a function of the frequency shift, extracted from two signals measured using two different field gradients: (a) $\nabla B = -13$ $\upmu$T/cm, (b) $\nabla B = -0.6$ $\upmu$T/cm. Both signals contain two modes which are indicated in green (mode 1) and magenta (mode 2). One can see a linear dependence of $\varOmega ^2$ on $\delta _{\omega }$. Both signals have been measured with $B_{\text{ RF }}=297$ nT and temperature $T=0.58\,T_c$. **B** Mode offset position $\delta _{\omega }^0$ as a function of the field gradient. Solid lines are frequency shifts at cell ends calculated using the known field profile of the gradient coil and the cell size. Signals were measured with $B_{\text{ RF }}=298$ and 358 nT in the whole temperature range over which the low-temperature modes were visible. **C** Temperature dependence of mode slopes $S_{LT}$. Different symbols are used for signals measured with different RF-field amplitudes: ($\bullet$), ($\circ$), ($*$) correspond to $B_{\text{ RF }}=238,297,357$ nT, respectively. $\nabla B = -0.6$ $\upmu$T/cm. The two colors are the same as in the other panels for the two different modes

**Fig. 5:**
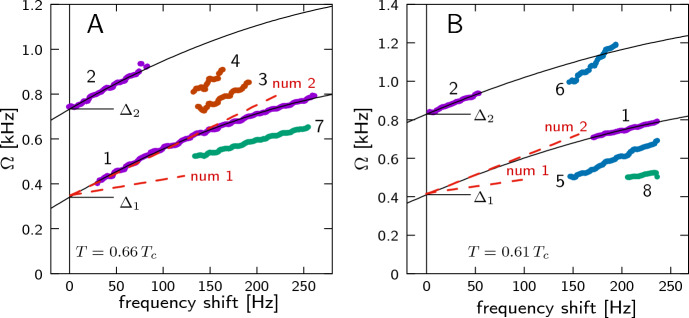
Examples of high-temperature modes. $B_{\text{ RF }}=297$ nT, $\nabla B=-0.9\,\upmu$T/cm. The frequency shift is counted from the point where mode 2 appears, as explained in the text. **A** $T=0.66\,T_c$, **B:** $T=0.61\,T_c$. Different modes are marked by numbers 1...8. Black lines is a quadratic fit of modes 1 and 2 with a single frequency shift dependence and different gaps. Red dashed lines “num 1” and “num 2” are results of numerical simulations in Ref. [22]

An important experimental parameter is the frequency shift $\delta _{\omega }= \omega - \gamma B$, the difference between pumping frequency $\omega$ which is always constant, and the Larmor frequency proportional to the magnetic field $\textbf{B}$, which changes during the experiments. The frequency shift varies across the sample because of the field inhomogeneity and the applied field gradient $\nabla \textbf{B}$. The inhomogeneity limits the accuracy of calibration of *B* and the absolute value of the frequency shift $\delta _{\omega }$. However, relative changes in the frequency shift are well-defined and accurate. Normally, we calibrate the magnetic field using NMR in liquid helium above $T_c$, and calculate the frequency shift using this calibration. We can only say that, at zero frequency shift condition, $\omega = \gamma B$ is valid in some region inside the sample chamber. Such frequency shift values are presented in Figs. 2, 3, and 4. For high-temperature modes (displayed in Fig. 5), we use a more specific calibration of the magnetic field: we assume that the field at which the modes appear corresponds to zero frequency shift at the exact position of the soliton (details will be given in the Results section).

## Results

Figure 2B displays a typical range of temperatures and frequency shifts, over which we can observe the HPD state. During each measurement, the magnetic field is swept continuously upwards, the HPD appears when the frequency shift crosses zero in some part of the cell and exists until a maximum frequency shift is reached. We found that this maximum value can be estimated in the following way. Using Leggett’s equations with radio-frequency field $B_{\text{ RF }}$ and including the Leggett–Takagi relaxation term [29], one can find the steady state that corresponds to the HPD. In the presence of Leggett–Takagi relaxation, the vector $\textbf{n}$ is deflected from the direction given by Eq. (3), in such a way that

5 $$\begin{aligned} n_x = \frac{\sqrt{15}}{4}\, \frac{\delta _{\omega }^2}{\varOmega _B^2}\, \frac{1}{\gamma B_{\text{ RF }}}\, \frac{1}{\tau } \end{aligned}$$

where $\tau$ is the Leggett–Takagi relaxation time which is of the order of the mean Bogolyubov quasiparticle relaxation time (we use values calculated in Ref. [30]). We do not know the exact conditions at which the HPD becomes unstable, but we can say that it should happen before the vector $\textbf{n}$ is rotated by 90 degrees, i.e., $n_x\approx 1$. Due to these approximations, we cannot have an exact expression, but only an estimation for the maximum frequency shift:

6 $$\begin{aligned} \delta _{\omega }{}_{\text{ max }} \approx \varOmega _B \sqrt{\gamma B_{\text{ RF }}\,\tau } \end{aligned}$$

In Fig. 2B, we display this estimation which matches the experimental results quite well when scaled by a factor of 0.58. Furthermore, the good agreement in the temperature dependence indicates that our thermometry corrections described in the Experiment section are realistic.

We find an HPD state at temperatures above approximately $0.5\,T_c$. Below this temperature the HPD is unstable because of catastrophic relaxation effects ([31, 32]). We distinguish two temperature ranges, below and above $T \approx 0.59\,T_c$, with completely different HPD oscillation modes. We call them “low-temperature” and “high-temperature” modes. At low temperatures and high frequency shifts we also observe chaotic motion of the HPD. This effect was studied numerically by Y. Bunkov in Ref. [33]. All these features can be seen in the spectrograms displayed in Fig. 3. The frequency spectrum is plotted as a function of time (bottom scale) and the frequency shift corresponding to the change of magnetic field in frequency units (upper scale). The left plot of Fig. 3A illustrates data recorded at $0.54\,T_c$ on the low-temperature modes and chaotic motion of the HPD; the right plot Fig. 3B presents data of the high-temperature modes recorded at $0.62\,T_c$. All modes are seen as symmetric side bands of the NMR frequency $\omega$. From the measured signals, we extract the mode frequencies $\varOmega$ as a function of the frequency shift $\delta _{\omega }$. We can see a clear dependence of the mode frequencies on the frequency shift in the presence of field inhomogeneity and different values of the applied field gradient. This indicates that the oscillation modes are localized.

**Low-temperature modes** are seen at $T < 0.59\,T_c$. We observe two different modes and their harmonics at $2\times$, $3\times$ the fundamental frequency (see the blue frame in Fig. 3A). The mode frequencies are proportional to the square root of the frequency shift and can be written as

7 $$\begin{aligned} \varOmega ^2 = S_{LT}\, (\delta _{\omega }-\delta _{\omega }^0), \end{aligned}$$

where the slope *S* and offset $\delta _{\omega }^0$ are mode-dependent parameters as seen for the data in Fig. 4A. We can assume that the low-temperature modes do not have any intrinsic frequency gap and the offset $\delta _{\omega }^0$ is determined only by the location inside the cell in the presence of a field gradient. We found that $\delta _{\omega }^0$ does not depend on temperature nor on the amplitude of the RF field, but it does depend on the field gradient in a way as if the modes are localized at the upper and lower ends of the experimental cell (Fig. 4B). The temperature and RF-field dependence of the slope parameter $S_{LT}$ of mode frequencies is illustrated in Fig. 4C.

**High-temperature modes** At higher temperature we observe a different set of oscillation modes (see the blue frame in Fig. 3B). In some rare cases they coexist with low-temperature modes, but usually there is a clear transition between these two regimes. There are many high-temperature modes, some are more common, while some appear only in a few measurements. Examples of two measurements are shown in Fig. 5, and different modes are marked by numbers 1...8.

Modes 1 and 2 are the most common ones, they appear in almost all measurements in the high-temperature regime. If we try to extrapolate their frequencies to zero, like we did with low-temperature modes, we find a big negative frequency shift which cannot be explained by field inhomogeneity. It means that the modes have some intrinsic frequency gap. We found that at the point where mode 2 becomes visible, a tiny but clear feature on the HPD signal can be observed ("oscillations start" line in Fig. 3B). We assume that this corresponds to the localization point of the modes, and the frequency shift should be measured from this value. We find that this point is always located in a random place inside the cell. (We can convert the magnetic field value to a position using the field gradient.) This allows us to determine the mode gaps $\varDelta _1$ and $\varDelta _2$. We see that the modes 1 and 2 have exactly the same frequency shift dependence, but there is a constant separation specified by the difference of $\varDelta _1$ and $\varDelta _2$ (see Fig. 5).

This information leads us to formulate our central conjecture: We reason that the high-temperature modes are oscillations of a $\vartheta$-soliton localized somewhere in the cell volume. Low-frequency oscillations of the $\vartheta$-soliton are calculated using 1D geometry as discussed in Ref. [22]. In our experiment, the soliton is comprised of a 3D circular membrane, in which case the radial part of oscillations should give an additional frequency gap

8 $$\begin{aligned} \varDelta _n = a_n C/r, \end{aligned}$$

where *C* is the wave velocity along the membrane, *r* is the radius, and $a_n$ denote the zeros of the Bessel function of the first kind. For the two first radial modes $a_1=2.405$, $a_2=5.520$. Note that Ref. [22] discusses another type of oscillation of the circular membrane, namely one in which the whole soliton is moving. These modes should have much larger frequency, but we do not find them in the experiment. In Fig. 5, the dashed lines marked as “num 1” and “num 2” display results of the 1D numerical simulation, given by the approximative formulas Eqs. (9) and (10) of Ref. [22], shifted by $\varDelta _1$. Apart from the gaps $\varDelta _1$ and $\varDelta _2$, we see that the measured modes 1 and 2 behave closely to the second calculated mode for the $\vartheta$-soliton. The behavior observed for modes 7 or 8 could correspond to the first mode, but it’s hard to conclude with certainty owing to the small visibility range of the mode in our experiment.

**Fig. 6:**
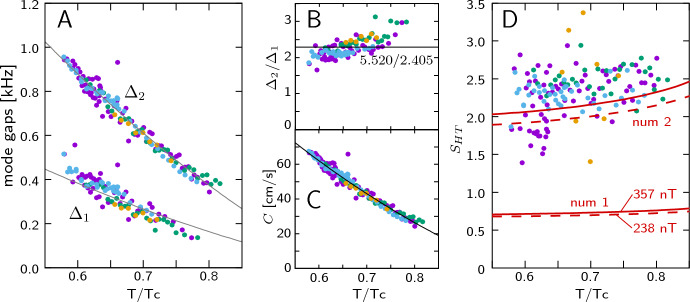
High-temperature modes 1 and 2 vs. *T*. Colors refer to different data sets measured in different cooling cycles using $B_{\text{ RF }}=238,297,357$ nT and $\nabla B =-0.9\,\upmu$T/cm. **A** Gaps $\varDelta _1$ and $\varDelta _2$ vs. temperature. The data are the same as in Fig. 2A but now with temperature correction. **B** Ratio $\varDelta _2/\varDelta _1$ for measurements where both modes exist. **C** Wave velocity *C* evaluated from $\varDelta _1$ and $\varDelta _2$ using Eq. (8). **D** Slope $S_{HT}$ of mode frequency $\varOmega$ vs. frequency shift $\delta _{\omega }$ in comparison with numerical simulations (lines “num1” and “num2”). Dashed lines are calculated using $B_{\text{ RF }}=238$ nT and the solid ones using $B_{\text{ RF }}=357$ nT

Further basic information on the high-temperature modes 1 and 2 is presented in Fig. 6. The mode gaps $\varDelta _1$ and $\varDelta _2$ are plotted as a function of temperature in Fig. 6A. These data are the same as in Fig. 2A, but with the temperature correction applied. We do not observe any noticeable dependence of the gaps on $B_{\text{ RF }}$ and $\nabla B$. In Fig. 6B, the ratio $\varDelta _2/\varDelta _1$ is plotted for measurements where both modes exist. The observed ratio is pretty close to the theoretical ratio given by $a_2/a_1 = 2.295$. The values deduced for the wave velocity $C = \varDelta _i r / a_n$ are depicted in Fig. 6C. In Fig. 6D we compare the linear term in the mode frequency $S_{HT} = \frac{d\varOmega }{d\delta _{\omega }}(\delta _{\omega }=0)$ with numerical simulation. We find good agreement with the calculated second mode of soliton oscillations, with only a weak dependence on temperature and $B_{\text{ RF }}$. The fact that we can have a separate temperature-dependent gap and an almost temperature-independent soliton mode supports our choice of $\delta _{\omega }=0$ for the frequency shift scale.

We leave the detailed analysis of the other high-temperature modes outside the scope of this paper. Modes 3 and 4 (see Fig. 5A) were quite common, but they existed only in a small frequency shift range. They look like the next two modes of $\vartheta$-soliton oscillations obtained in the numerical simulation, but at the moment we cannot confirm it. Modes 5 and 6 (see Fig. 5B) appeared in pairs with a frequency ratio of 2. They do not extrapolate to the same energy gap as modes 1 and 2, and probably they do not have a gap. Modes 7 and 8 were visible only in a few signals, which is unfortunate as they seem to bear resemblance with the first mode in our simulations. However, due to scarce information we could not analyze and classify them accurately.

## Conclusions

In this work, we measured the response of the HPD state in an about 10 kHz band around the NMR frequency and could make interesting observations: new oscillation modes localized near the cell walls, additional modes in the bulk volume of the HPD that we attribute to oscillations of $\vartheta$-solitons, and chaotic motion at high frequency shifts. We also estimated the maximum frequency shift at which the HPD state is stable and found that our experimental result matches our estimations based on Leggett–Takagi relaxation.

The richness of the observed soliton oscillation modes makes the most interesting finding of our experiments. Our work shows that, overall, the observed mode frequencies match with numerical simulations, but there are still more questions than answers: how these modes are excited, how the solitons are created, how to classify them and explain all the observed modes. Further investigation is certainly needed, in experiment, theory, and numerical simulations. It would be interesting to study how solitons are created at different magnetic field ramping rates, to isolate a single soliton by tuning experimental conditions without destroying the HPD, to populate certain modes by using additional RF-field excitation pulses, etc. It would be important to study how the creation of a soliton is affected by the magnetic field profile and sweeping rate. Altogether, the soliton dynamics would be a sophisticated and quite fundamental problem for further investigations.

## Data Availability

Our measured data are available on 10.5281/zenodo.8431264. It includes oscilloscope records, spectrograms, and extracted oscillation modes for about six hundred measurements.
